# 3-(3-Chloro­phenyl­sulfon­yl)-2,5-dimethyl-1-benzofuran

**DOI:** 10.1107/S1600536811035720

**Published:** 2011-09-14

**Authors:** Pil Ja Seo, Hong Dae Choi, Byeng Wha Son, Uk Lee

**Affiliations:** a Department of Chemistry, Dongeui University, San 24 Kaya-dong Busanjin-gu, Busan 614-714, Republic of Korea; b Department of Chemistry, Pukyong National University, 599-1 Daeyeon 3-dong, Nam-gu, Busan 608-737, Republic of Korea

## Abstract

In the title molecule, C_16_H_13_ClO_3_S, the 3-chloro­phenyl ring makes a dihedral angle of 76.30 (5)° with the mean plane of the benzofuran fragment. In the crystal, pairs of inter­molecular C—H⋯π inter­actions link the mol­ecules into inversion dimers.

## Related literature

For the pharmacological activity of benzofuran compounds, see: Aslam *et al.* (2009 ▶); Galal *et al.* (2009 ▶); Khan *et al.* (2005 ▶). For natural products with benzofuran rings, see: Akgul & Anil (2003 ▶); Soekamto *et al.* (2003 ▶). For structural studies of 3-(4-chloro­phenyl­sulfon­yl)-2-methyl-1-benzofuran derivatives, see: Choi *et al.* (2010 ▶, 2011 ▶).
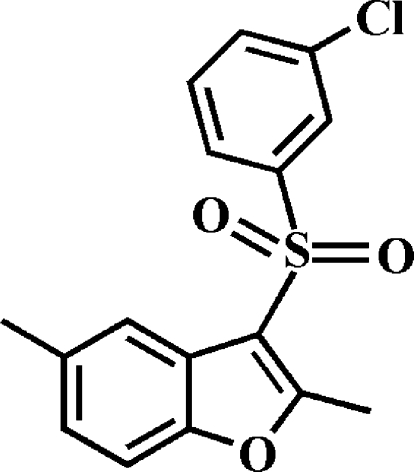


## Experimental

### 

#### Crystal data

C_16_H_13_ClO_3_S
*M* *_r_* = 320.77
Triclinic, 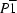

*a* = 8.1000 (4) Å
*b* = 8.8622 (5) Å
*c* = 10.6898 (6) Å
α = 84.023 (3)°
β = 81.514 (3)°
γ = 72.378 (3)°
*V* = 721.90 (7) Å^3^
*Z* = 2
Mo *K*α radiation
μ = 0.42 mm^−1^
*T* = 173 K
0.28 × 0.27 × 0.19 mm

#### Data collection

Bruker SMART APEXII CCD diffractometer
Absorption correction: multi-scan (*SADABS*; Bruker, 2009 ▶) *T*_min_ = 0.579, *T*_max_ = 0.746
13538 measured reflections
3622 independent reflections
2951 reflections with *I* > 2σ(*I*)
*R*_int_ = 0.051

#### Refinement

*R*[*F* ^2^ > 2σ(*F* ^2^)] = 0.040
*wR*(*F* ^2^) = 0.110
*S* = 1.05
3622 reflections
192 parameters
H-atom parameters constrained
Δρ_max_ = 0.39 e Å^−3^
Δρ_min_ = −0.40 e Å^−3^

### 

Data collection: *APEX2* (Bruker, 2009 ▶); cell refinement: *SAINT* (Bruker, 2009 ▶); data reduction: *SAINT*; program(s) used to solve structure: *SHELXS97* (Sheldrick, 2008 ▶); program(s) used to refine structure: *SHELXL97* (Sheldrick, 2008 ▶); molecular graphics: *ORTEP-3* (Farrugia, 1997 ▶) and *DIAMOND* (Brandenburg, 1998 ▶); software used to prepare material for publication: *SHELXL97*.

## Supplementary Material

Crystal structure: contains datablock(s) I. DOI: 10.1107/S1600536811035720/qm2026sup1.cif

Structure factors: contains datablock(s) I. DOI: 10.1107/S1600536811035720/qm2026Isup2.hkl

Supplementary material file. DOI: 10.1107/S1600536811035720/qm2026Isup3.cml

Additional supplementary materials:  crystallographic information; 3D view; checkCIF report

## Figures and Tables

**Table 1:** Hydrogen-bond geometry (Å, °) *Cg* is the centroid of the C2–C7 benzene ring.

*D*—H⋯*A*	*D*—H	H⋯*A*	*D*⋯*A*	*D*—H⋯*A*
C9—H9*C*⋯*Cg*^i^	0.98	2.73	3.663 (2)	159

Symmetry code: (i) 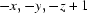.

**Fig. 1.**
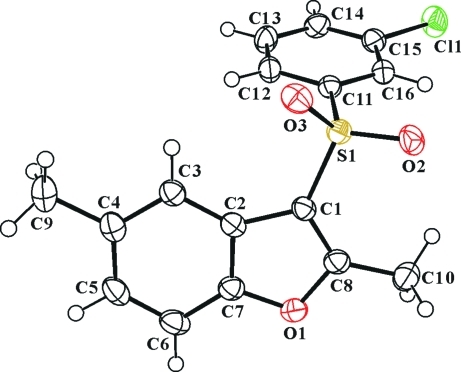
The molecular structure of the title compound with the atom numbering scheme. Displacement ellipsoids are drawn at the 50% probability level. H atoms are presented as small spheres of arbitrary radius.

**Fig. 2.**
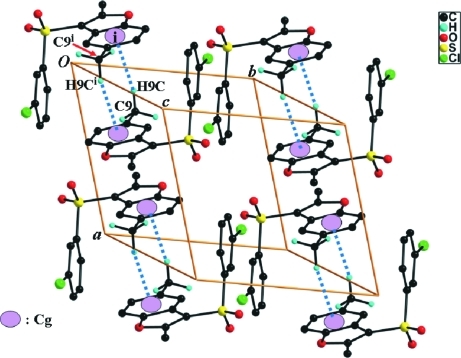
A view of the C—H···π interactions (dotted lines) in the crystal structure of the title compound. [Symmetry codes: (i) - x, - y, - z + 1.]

## supplementary crystallographic information

### Comment

Recently, many compounds containing a benzofuran ring system have drawn much attention owing to their valuable pharmacological properties such as antibacterial and antifungal, antitumor and antiviral, and antimicrobial activities (Aslam *et al.*, 2009, Galal *et al.*, 2009, Khan *et al.*, 2005). These benzofuran derivatives occur in a wide range of natural products (Akgul & Anil, 2003; Soekamto *et al.*, 2003). As a part of our ongoing study of the substituent effect on the solid state structures of 3-(4-chlorophenylsulfonyl)-2-methyl-1-benzofuran analogues (Choi *et al.*, 2010, 2011), we report herein the crystal structure of the title compound.

In the title molecule (Fig. 1), the benzofuran unit is essentially planar, with a mean deviation of 0.004 (1) Å from the least-squares plane defined by the nine constituent atoms. The dihedral angle formed by the 3-chlorophenyl ring and the mean plane of the benzofuran fragment is 76.30 (5)° . The crystal packing (Fig.2) is stabilized by intermolecular C—H···π interactions between a methyl H atom and the benzene ring (Table 1; C9—H9C···Cg^i^, Cg is the centroid of the C2–C7 benzene ring).

### Experimental

77% 3-chloroperoxybenzoic acid (514 mg, 2.3 mmol) was added in small portions to a stirred solution of 3-(3-chlorophenylsulfanyl)-2,5-dimethyl-1-benzofuran (317 mg, 1.1 mmol) in dichloromethane (30 mL) at 273 K. After being stirred at room temperature for 4h, the mixture was washed with saturated sodium bicarbonate solution and the organic layer was separated, dried over magnesium sulfate, filtered and concentrated at reduced pressure. The residue was purified by column chromatography (hexane–ethyl acetate, 4:1 v/v) to afford the title compound as a colorless solid [yield 68%, m.p. 396–397 K; Rf = 0.75 (hexane–ethyl acetate, 4:1 v/v)]. Single crystals suitable for X-ray diffraction were prepared by slow evaporation of a solution of the title compound in acetone at room temperature.

### Refinement

All H atoms were positioned geometrically and refined using a riding model, with C—H = 0.95 Å for aryl and 0.99 Å for methyl H atoms. *U*_iso_(H)  = 1.2*U*_eq_(C) for aryl and 1.5*U*_eq_(C) for methyl H atoms.

### Crystal data

**Table:**

C_16_H_13_ClO_3_S	*Z* = 2
*M**_r_* = 320.77	*F*(000) = 332
Triclinic, *P*1	*D*_x_ = 1.476 Mg m^−^^3^
Hall symbol: -P 1	Mo *K*α radiation, λ = 0.71073 Å
*a* = 8.1000 (4) Å	Cell parameters from 5186 reflections
*b* = 8.8622 (5) Å	θ = 2.4–27.9°
*c* = 10.6898 (6) Å	µ = 0.42 mm^−^^1^
α = 84.023 (3)°	*T* = 173 K
β = 81.514 (3)°	Block, colourless
γ = 72.378 (3)°	0.28 × 0.27 × 0.19 mm
*V* = 721.90 (7) Å^3^	

### Data collection

**Table:**

Bruker SMART APEXII CCD diffractometer	3622 independent reflections
Radiation source: rotating anode	2951 reflections with *I* > 2σ(*I*)
graphite multilayer	*R*_int_ = 0.051
Detector resolution: 10.0 pixels mm^-1^	θ_max_ = 28.4°, θ_min_ = 1.9°
φ and ω scans	*h* = −10→10
Absorption correction: multi-scan (*SADABS*; Bruker, 2009)	*k* = −11→11
*T*_min_ = 0.579, *T*_max_ = 0.746	*l* = −14→14
13538 measured reflections	

### Refinement

**Table:**

Refinement on *F*^2^	Primary atom site location: structure-invariant direct methods
Least-squares matrix: full	Secondary atom site location: difference Fourier map
*R*[*F*^2^ > 2σ(*F*^2^)] = 0.040	Hydrogen site location: difference Fourier map
*wR*(*F*^2^) = 0.110	H-atom parameters constrained
*S* = 1.05	*w* = 1/[σ^2^(*F*_o_^2^) + (0.0497*P*)^2^ + 0.243*P*] where *P* = (*F*_o_^2^ + 2*F*_c_^2^)/3
3622 reflections	(Δ/σ)_max_ < 0.001
192 parameters	Δρ_max_ = 0.39 e Å^−^^3^
0 restraints	Δρ_min_ = −0.40 e Å^−^^3^

### Special details

**Table:**

Geometry. All esds (except the esd in the dihedral angle between two l.s. planes) are estimated using the full covariance matrix. The cell esds are taken into account individually in the estimation of esds in distances, angles and torsion angles; correlations between esds in cell parameters are only used when they are defined by crystal symmetry. An approximate (isotropic) treatment of cell esds is used for estimating esds involving l.s. planes.
Refinement. Refinement of F^2^ against ALL reflections. The weighted R-factor wR and goodness of fit S are based on F^2^, conventional R-factors R are based on F, with F set to zero for negative F^2^. The threshold expression of F^2^ > 2sigma(F^2^) is used only for calculating R-factors(gt) etc. and is not relevant to the choice of reflections for refinement. R-factors based on F^2^ are statistically about twice as large as those based on F, and R- factors based on ALL data will be even larger.

### Fractional atomic coordinates and isotropic or equivalent isotropic displacement parameters (Å^2^)

**Table:**

	*x*	*y*	*z*	*U*_iso_*/*U*_eq_	
S1	0.33167 (5)	0.43504 (5)	0.29715 (4)	0.02475 (13)	
Cl1	−0.08568 (7)	0.78553 (6)	−0.04210 (5)	0.04248 (16)	
O1	0.49244 (16)	−0.00637 (14)	0.20581 (12)	0.0310 (3)	
O2	0.45023 (15)	0.49763 (14)	0.20924 (13)	0.0328 (3)	
O3	0.31247 (17)	0.46293 (15)	0.42888 (12)	0.0324 (3)	
C1	0.3859 (2)	0.23236 (19)	0.28520 (16)	0.0239 (3)	
C2	0.3302 (2)	0.12036 (19)	0.37751 (16)	0.0242 (3)	
C3	0.2342 (2)	0.1260 (2)	0.49708 (16)	0.0272 (4)	
H3	0.1872	0.2233	0.5372	0.033*	
C4	0.2087 (2)	−0.0133 (2)	0.55658 (17)	0.0298 (4)	
C5	0.2811 (2)	−0.1561 (2)	0.49675 (19)	0.0342 (4)	
H5	0.2626	−0.2505	0.5389	0.041*	
C6	0.3782 (2)	−0.1647 (2)	0.3793 (2)	0.0341 (4)	
H6	0.4273	−0.2621	0.3395	0.041*	
C7	0.3998 (2)	−0.0244 (2)	0.32300 (17)	0.0274 (4)	
C8	0.4815 (2)	0.1509 (2)	0.18512 (17)	0.0273 (4)	
C9	0.1031 (3)	−0.0119 (3)	0.68477 (19)	0.0404 (5)	
H9A	0.1140	0.0730	0.7319	0.061*	
H9B	0.1462	−0.1143	0.7316	0.061*	
H9C	−0.0198	0.0063	0.6744	0.061*	
C10	0.5727 (2)	0.1939 (2)	0.06278 (18)	0.0367 (4)	
H10A	0.5343	0.3093	0.0459	0.055*	
H10B	0.5455	0.1426	−0.0052	0.055*	
H10C	0.6989	0.1584	0.0665	0.055*	
C11	0.1232 (2)	0.51080 (19)	0.24554 (15)	0.0241 (3)	
C12	−0.0193 (2)	0.4771 (2)	0.31850 (17)	0.0287 (4)	
H12	−0.0056	0.4149	0.3963	0.034*	
C13	−0.1810 (2)	0.5353 (2)	0.27665 (18)	0.0326 (4)	
H13	−0.2789	0.5109	0.3250	0.039*	
C14	−0.2017 (2)	0.6286 (2)	0.16502 (17)	0.0313 (4)	
H14	−0.3133	0.6683	0.1362	0.038*	
C15	−0.0585 (2)	0.6636 (2)	0.09564 (17)	0.0290 (4)	
C16	0.1054 (2)	0.6038 (2)	0.13328 (16)	0.0277 (4)	
H16	0.2037	0.6259	0.0835	0.033*	

### Atomic displacement parameters (Å^2^)

**Table:**

	*U*^11^	*U*^22^	*U*^33^	*U*^12^	*U*^13^	*U*^23^
S1	0.0225 (2)	0.0215 (2)	0.0316 (2)	−0.00858 (15)	−0.00493 (16)	0.00078 (16)
Cl1	0.0428 (3)	0.0451 (3)	0.0349 (3)	−0.0092 (2)	−0.0093 (2)	0.0141 (2)
O1	0.0274 (6)	0.0253 (6)	0.0376 (7)	−0.0053 (5)	0.0013 (5)	−0.0050 (5)
O2	0.0245 (6)	0.0294 (7)	0.0460 (8)	−0.0130 (5)	−0.0032 (5)	0.0051 (5)
O3	0.0383 (7)	0.0286 (6)	0.0335 (7)	−0.0114 (5)	−0.0106 (5)	−0.0027 (5)
C1	0.0210 (7)	0.0221 (7)	0.0294 (8)	−0.0071 (6)	−0.0048 (6)	0.0008 (6)
C2	0.0198 (7)	0.0225 (8)	0.0315 (9)	−0.0071 (6)	−0.0072 (6)	0.0010 (6)
C3	0.0259 (8)	0.0260 (8)	0.0300 (9)	−0.0075 (6)	−0.0059 (7)	0.0003 (7)
C4	0.0257 (8)	0.0330 (9)	0.0325 (9)	−0.0115 (7)	−0.0093 (7)	0.0067 (7)
C5	0.0314 (9)	0.0254 (9)	0.0469 (11)	−0.0114 (7)	−0.0093 (8)	0.0083 (8)
C6	0.0310 (9)	0.0222 (8)	0.0495 (12)	−0.0075 (7)	−0.0062 (8)	−0.0024 (8)
C7	0.0219 (8)	0.0256 (8)	0.0343 (9)	−0.0062 (6)	−0.0040 (7)	−0.0008 (7)
C8	0.0218 (8)	0.0256 (8)	0.0339 (9)	−0.0059 (6)	−0.0053 (7)	0.0001 (7)
C9	0.0420 (11)	0.0465 (11)	0.0343 (10)	−0.0199 (9)	−0.0036 (8)	0.0096 (9)
C10	0.0323 (9)	0.0402 (10)	0.0331 (10)	−0.0079 (8)	0.0025 (8)	0.0003 (8)
C11	0.0222 (8)	0.0223 (8)	0.0273 (8)	−0.0061 (6)	−0.0024 (6)	−0.0010 (6)
C12	0.0264 (8)	0.0293 (9)	0.0288 (9)	−0.0083 (7)	−0.0019 (7)	0.0047 (7)
C13	0.0237 (8)	0.0375 (10)	0.0349 (10)	−0.0095 (7)	0.0001 (7)	0.0023 (8)
C14	0.0241 (8)	0.0338 (9)	0.0330 (9)	−0.0034 (7)	−0.0054 (7)	−0.0011 (7)
C15	0.0307 (9)	0.0263 (8)	0.0270 (9)	−0.0046 (7)	−0.0045 (7)	0.0016 (7)
C16	0.0264 (8)	0.0257 (8)	0.0298 (9)	−0.0079 (7)	−0.0008 (7)	0.0012 (7)

### Geometric parameters (Å, °)

**Table:**

S1—O3	1.4328 (13)	C6—H6	0.9500
S1—O2	1.4355 (12)	C8—C10	1.478 (2)
S1—C1	1.7289 (17)	C9—H9A	0.9800
S1—C11	1.7659 (17)	C9—H9B	0.9800
Cl1—C15	1.7334 (18)	C9—H9C	0.9800
O1—C8	1.366 (2)	C10—H10A	0.9800
O1—C7	1.383 (2)	C10—H10B	0.9800
C1—C8	1.357 (2)	C10—H10C	0.9800
C1—C2	1.449 (2)	C11—C16	1.383 (2)
C2—C7	1.387 (2)	C11—C12	1.387 (2)
C2—C3	1.392 (2)	C12—C13	1.377 (3)
C3—C4	1.386 (2)	C12—H12	0.9500
C3—H3	0.9500	C13—C14	1.380 (3)
C4—C5	1.401 (3)	C13—H13	0.9500
C4—C9	1.504 (3)	C14—C15	1.381 (2)
C5—C6	1.376 (3)	C14—H14	0.9500
C5—H5	0.9500	C15—C16	1.376 (2)
C6—C7	1.373 (3)	C16—H16	0.9500
			
O3—S1—O2	119.46 (8)	C4—C9—H9A	109.5
O3—S1—C1	107.56 (8)	C4—C9—H9B	109.5
O2—S1—C1	109.13 (8)	H9A—C9—H9B	109.5
O3—S1—C11	107.49 (8)	C4—C9—H9C	109.5
O2—S1—C11	107.47 (8)	H9A—C9—H9C	109.5
C1—S1—C11	104.80 (8)	H9B—C9—H9C	109.5
C8—O1—C7	106.97 (13)	C8—C10—H10A	109.5
C8—C1—C2	107.83 (15)	C8—C10—H10B	109.5
C8—C1—S1	126.08 (13)	H10A—C10—H10B	109.5
C2—C1—S1	126.02 (12)	C8—C10—H10C	109.5
C7—C2—C3	119.05 (16)	H10A—C10—H10C	109.5
C7—C2—C1	104.37 (15)	H10B—C10—H10C	109.5
C3—C2—C1	136.58 (16)	C16—C11—C12	121.36 (16)
C4—C3—C2	118.71 (16)	C16—C11—S1	119.09 (12)
C4—C3—H3	120.6	C12—C11—S1	119.55 (13)
C2—C3—H3	120.6	C13—C12—C11	119.07 (16)
C3—C4—C5	119.84 (16)	C13—C12—H12	120.5
C3—C4—C9	120.42 (17)	C11—C12—H12	120.5
C5—C4—C9	119.74 (17)	C12—C13—C14	120.52 (16)
C6—C5—C4	122.43 (17)	C12—C13—H13	119.7
C6—C5—H5	118.8	C14—C13—H13	119.7
C4—C5—H5	118.8	C13—C14—C15	119.29 (17)
C7—C6—C5	116.06 (17)	C13—C14—H14	120.4
C7—C6—H6	122.0	C15—C14—H14	120.4
C5—C6—H6	122.0	C16—C15—C14	121.53 (16)
C6—C7—O1	125.54 (16)	C16—C15—Cl1	119.16 (14)
C6—C7—C2	123.90 (16)	C14—C15—Cl1	119.31 (14)
O1—C7—C2	110.56 (15)	C15—C16—C11	118.19 (16)
C1—C8—O1	110.26 (15)	C15—C16—H16	120.9
C1—C8—C10	134.82 (17)	C11—C16—H16	120.9
O1—C8—C10	114.92 (15)		
			
O3—S1—C1—C8	−151.41 (16)	C1—C2—C7—O1	0.08 (19)
O2—S1—C1—C8	−20.43 (19)	C2—C1—C8—O1	−0.2 (2)
C11—S1—C1—C8	94.41 (17)	S1—C1—C8—O1	−177.39 (12)
O3—S1—C1—C2	31.89 (17)	C2—C1—C8—C10	179.7 (2)
O2—S1—C1—C2	162.86 (14)	S1—C1—C8—C10	2.5 (3)
C11—S1—C1—C2	−82.29 (16)	C7—O1—C8—C1	0.2 (2)
C8—C1—C2—C7	0.06 (19)	C7—O1—C8—C10	−179.65 (15)
S1—C1—C2—C7	177.27 (13)	O3—S1—C11—C16	133.45 (14)
C8—C1—C2—C3	179.0 (2)	O2—S1—C11—C16	3.68 (16)
S1—C1—C2—C3	−3.8 (3)	C1—S1—C11—C16	−112.32 (14)
C7—C2—C3—C4	−1.3 (3)	O3—S1—C11—C12	−46.03 (16)
C1—C2—C3—C4	179.93 (18)	O2—S1—C11—C12	−175.80 (14)
C2—C3—C4—C5	0.9 (3)	C1—S1—C11—C12	68.19 (16)
C2—C3—C4—C9	−178.90 (17)	C16—C11—C12—C13	1.3 (3)
C3—C4—C5—C6	−0.1 (3)	S1—C11—C12—C13	−179.25 (14)
C9—C4—C5—C6	179.68 (18)	C11—C12—C13—C14	−1.4 (3)
C4—C5—C6—C7	−0.3 (3)	C12—C13—C14—C15	−0.2 (3)
C5—C6—C7—O1	179.85 (17)	C13—C14—C15—C16	1.9 (3)
C5—C6—C7—C2	−0.1 (3)	C13—C14—C15—Cl1	−178.12 (15)
C8—O1—C7—C6	179.81 (18)	C14—C15—C16—C11	−2.0 (3)
C8—O1—C7—C2	−0.20 (19)	Cl1—C15—C16—C11	178.02 (13)
C3—C2—C7—C6	0.9 (3)	C12—C11—C16—C15	0.4 (3)
C1—C2—C7—C6	−179.93 (17)	S1—C11—C16—C15	−179.09 (13)
C3—C2—C7—O1	−179.07 (15)		

### Hydrogen-bond geometry (Å, °)

**Table:**

Cg is the centroid of the C2–C7 benzene ring.

**Table:**

*D*—H···*A*	*D*—H	H···*A*	*D*···*A*	*D*—H···*A*
C9—H9C···Cg^i^	0.98	2.73	3.663 (2)	159.

Symmetry codes: (i) −*x*, −*y*, −*z*+1.
